# A Treasure Trove of Urban Microbial Diversity: Community and Diversity Characteristics of Urban Ancient *Ginkgo biloba* Rhizosphere Microorganisms in Shanghai

**DOI:** 10.3390/jof10100720

**Published:** 2024-10-16

**Authors:** Jieying Mao, Qiong Wang, Yaying Yang, Feng Pan, Ziwei Zou, Xiaona Su, Yi Wang, Wei Liu, Yaohua Tang

**Affiliations:** 1 Jiangxi Provincial Key Laboratory of Conservation Biology, Nanchang 330045, China; 15757555875@163.com (J.M.); wangqiong881004@163.com (Q.W.); yangyy0725@163.com (Y.Y.); zzwei0911@163.com (Z.Z.); 13561584223@163.com (X.S.); 18207000523@163.com (Y.W.); 2 School of Art and Landscape, College of Forestry, Jiangxi Agricultural University, Nanchang 330045, China; 3 Ningbo Yongneng Biomass Energy Development Co., Ltd., Ningbo 315000, China; 4 Ganzhou Vegetable and Flower Rrerarch Institute, Ganzhou 341413, China; 5 Jiangxi Academy of Water Science and Engineering, Nanchang 330029, China; panfeng_1987@163.com; 6 Shanghai Municipal Landscape Management and Guidance Station, Shanghai 200020, China

**Keywords:** ancient *Ginkgo biloba*, phospholipid fatty acid, high-throughput sequencing technology, microorganism, arbuscular mycorrhizal fungi, community characteristics

## Abstract

Rapid urbanization has exerted immense pressure on urban environments, severely constraining the growth of ancient trees. The growth of ancient trees is closely linked to the microbial communities in their rhizospheres, and studying their community characteristics may provide new insights into promoting the growth and rejuvenation of ancient trees. In this study, the rhizosphere soil and root systems of ancient *Ginkgo biloba* trees (approximately 200 years old) and adult *G. biloba* trees (approximately 50 years old) in Shanghai were selected as research subjects. Phospholipid fatty acid (PLFA) analysis and high-throughput sequencing were employed to investigate the diversity of microbial communities in the *G. biloba* rhizosphere. The results indicated that the 19 PLFA species selected to characterize the soil microbial community structure and biomass were present in the rhizosphere soil of both ancient and adult *G. biloba* trees. However, the total microbial biomass and the microbial biomass in the rhizosphere soil of ancient *G. biloba* were lower than the microbial biomass in the rhizosphere soil of adult *G. biloba*. The biomasses of Gram-negative bacteria (G^−^), arbuscular mycorrhizal fungi (AMF), and protozoans (P) were significantly different. Total phosphorus, organic matter, and pH may be the key factors influencing the soil microbial community in the rhizosphere zone of ancient *G. biloba*. An in-depth study of AMF showed that the roots and rhizosphere soil of *G. biloba* contained abundant AMF resources, which were assigned to 224 virtual taxa using the MaarjAM reference database, belonging to four orders, ten families, and nineteen genera. The first and second most dominant genera were *Glomus* and *Paraglomus*, respectively. *Archaeospora* and *Ambispora* were more dominant in the rhizosphere than the roots. Furthermore, the abundance of live AMF was significantly higher in ancient *G. biloba* than in adult *G. biloba*. Therefore, future research should focus on the improvement of soil environmental characteristics and the identification and cultivation of indigenous dominant AMF in the rhizosphere of ancient *G. biloba*, aiming for their effective application in the rejuvenation of ancient trees.

## 1. Introduction

According to China’s National Technical Regulations for the Census of Ancient and Famous Trees, published in 2001, ancient trees are defined as trees that are 100 years old and older [1]. These trees carry important historical memories and research value, making them a valuable natural and cultural heritage resource [2]. However, the vigor of ancient trees gradually weakens after a long period of growth. Natural disasters, pests, and diseases are likely to cause harm to ancient trees. Moreover, old trees that grow in cities have an even less optimistic outlook. *Ginkgo biloba* is an endemic tree species in China that only blossoms and bears fruit after about 20 years [3]. The development of flowers occurs when *G. biloba* reaches adulthood [4]. With a large population, *G. biloba* is an important ancient and famous tree resource in China. *G. biloba* is the most abundant ancient tree species in Shanghai [5]. However, according to the Shanghai Municipal Administration of Greening and Amenities, approximately one-third of ancient *G. biloba* in Shanghai exhibit signs of poor growth owing to natural and anthropogenic influences.

Soil quality is among the most important factors affecting the growth of ancient trees. Problems such as soil compaction, heavy metal pollution, mineral nutrient scarcity, and imbalances in rationing affect the health of ancient trees, resulting in the slow growth of their root systems and an insufficient supply of nutrients. The most active components of the soil are microorganisms, which can interact with the soil. The soil microbiota is also an important indicator of soil quality [6]. Therefore, there is a strong link between the soil microbiota and the growth of ancient trees. However, studies have shown that there are significant differences in the structure of the microbiota between the rhizosphere and non-rhizosphere soils of *G. biloba*, and the profile of soil fungi is complex and inconsistent between these soils [7].

Arbuscular mycorrhizal fungi (AMF) are a group of endophytic fungi widely distributed in terrestrial ecosystems [8]. AMF are present in the soil as spores and mycelia. AMF account for the largest proportion of the fungal biomass in soil and directly affect the microbial community structure through mycelial secretions that cause changes in plant root secretions [9]. Previous studies have confirmed that AMF infesting the root system of *G. biloba* is a common phenomenon that forms a mutually beneficial symbiotic system with the host. Fontana [10] found that *G. biloba* on the campus of the Università degli Studi di Torino, Italy, exhibited numerous intracellular mycelia. Wu and Wei [11] found that the development of clumped mycorrhizae in grafted *G. biloba* seedlings was significantly better than that seen in live seedlings grown through seed germination. Yuan et al. [12] determined that the AMF of *G. biloba* growing on artificial coasts exhibited a P-type structure. In a symbiotic system formed with the host plant, AMF can absorb mineral nutrients through mycelial extensions into soil crevices that are inaccessible to the plant root system for transfer to the host plant [13,14]. Additionally, AMF have various beneficial effects on soil, including improving the physical structure, stabilizing organic carbon, activating nutrients, and maintaining pore space [15,16].

Tong et al. [17] applied exogenous mycorrhizal fungi to ancient *G. biloba* in Shanghai and found that AMF inoculation significantly promoted the growth of ancient trees, with enhanced soil fertility detected four years later. This suggests that mycorrhizal fungi can promote the rejuvenation of ancient *G. biloba*. In fact, indigenous mycorrhizal fungi in the rhizosphere soil of plants may be more adaptable to the soil environment and function more efficiently than exogenous mycorrhizal fungi because they have a closer relationship with the host plants [18]. Therefore, exploring the diversity of microbial communities, especially AMF communities, may provide a new direction for promoting the growth of ancient *G. biloba* and for salvage and rejuvenation.

After hundreds or even thousands of years of growth, ancient *G. biloba* may have a more stable microbial community in its rhizosphere than younger adult *G. biloba*. In this study, it was hypothesized that (1) ancient *G. biloba* trees accumulate more microbial species and numbers in the rhizosphere soil than younger adult *G. biloba*; and (2) AMF are present in both the roots and rhizosphere soils of *G. biloba* but may differ considerably in terms of species and number between ancient and younger adult *G. biloba*.

To test these hypotheses, phospholipid fatty acid (PLFA) technology was employed to detect microbial taxa in the rhizosphere soil of ancient and younger adult *G. biloba* in Shanghai. High-throughput sequencing technology was utilized to reveal the community composition and diversity of AMF in the rhizosphere of *G. biloba* in depth. It is hoped that this study will provide a research basis for identifying microbial taxa in the rhizosphere soils of ancient *G. biloba*, as well as for the preparation of mycorrhizal agents to promote the growth and rejuvenation of ancient trees.

## 2. Materials and Methods

### 2.1. Site and Experimental Design

**Figure 1:**
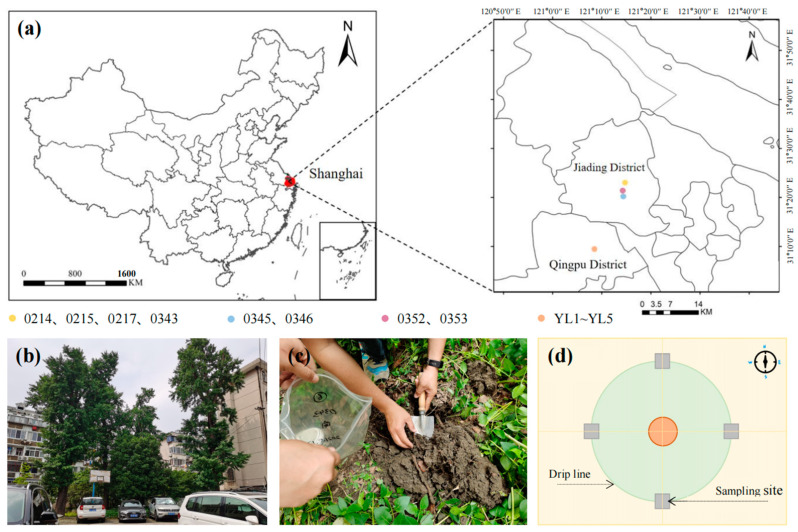
Sampling positions and process. (**a**) Map of study sites; (**b**) sampling sites; (**c**) sampling process; and (**d**) sampling diagram.

The sampling sites were located in the Jiading District and Qingpu District (31°9′14′′ N–31°23′3′′ N, 121°8′35′′ E–121°27′5′′ E) of Shanghai, China (Figure 1). The region has a northern subtropical monsoon climate with warm weather and ample precipitation. The average annual temperature is 17.7 °C, and precipitation is concentrated from April through October, with an average annual precipitation of 1726 mm.

To explore the symbiosis between AMF and *G. biloba*, two types of *G. biloba* were selected for the study: eight ancient *G. biloba* approximately 200 years old, and five adult *G. biloba* approximately 50 years old. The ages of ancient *G. biloba* trees were determined according to the times recorded on the protection plates for ancient and famous trees established by the Shanghai Municipal Administration of Greening and Amenities, which were obtained through a literature review [19]. The ages of adult *G. biloba* trees were estimated based on the landscaping projects implemented by the Shanghai Municipal Administration of Greening and Amenities at that time. None of these trees were treated with mycorrhizal fungicides. Fieldwork was performed on 17 May 2021 and 15 September 2022, and the relevant information about each tree was recorded (Table 1). The drip lines of *G. biloba* were located in four directions: east, west, south, and north. During sample collection, 150 g of mixed soil and roots were collected to a depth of approximately 30 cm. Samples from all four directions were placed on a newspaper sheet to remove plant debris and stones. The samples were then mixed thoroughly in a sealing bag to form a composite sample, and this process was repeated three times for each tree. To prevent contamination, the shovel was cleaned to remove soil particles and disinfected with 70% ethanol before and after digging, and sterile gloves were utilized during the removal of plant debris and stones.

For sample preservation, it was necessary to separate the roots from the soil in each sample and rinse the root surface. The samples were labeled as the roots of ancient *G. biloba* (GYXG), the rhizosphere soil of ancient *G. biloba* (GYXT), the roots of adult *G. biloba* (XYXG), and the rhizosphere soil of adult *G. biloba* (XYXT). Each soil sample was equally divided into three parts, one of which was given to the South China Botanical Garden for the PLFA assay based on the methodology referenced from Bossio et al. [20] and analyzed using the MIDI Sherlock^®^ Microbial Identification System 6.0. Another part, together with the roots of *G. biloba*, was sent to Novogene for high-throughput sequencing. As the samples were not immediately available for PLFA and high-throughput sequencing assays, they were stored temporarily at −20 °C. The final part was used to determine the chemical properties, stored at room temperature (20–25 °C), sieved (2 mm), spread flat in the laboratory, and allowed to dry naturally.

### 2.2. Experimental Methods

#### 2.2.1. Soil Chemical Analyses

The soil was digested using the HClO_4_-H_2_SO_4_ decoction method before the analysis of the total nitrogen (TN), total phosphorus (TP), and total potassium (TK) contents. The TN was analyzed using a flow analyzer (AutoAnalyzer III, Bran + Luebbe GmbH, Hamburg, Germany). The TP was measured using the molybdenum antimony anti-colorimetric method [21], and the TK was determined using a flame photometer (FP640, Jingke, Shanghai, China). The potassium dichromate-potassium external heating method was employed to determine the organic matter (OM) content. The soil pH was measured using a pH meter (PB-10, Sartorius AG, Göttingen, Germany), and the soil conductivity was measured using a conductivity meter (DDS-307, Yidian Corporation, Shanghai, China). Each soil sample was replicated three times with a blank control.

#### 2.2.2. PLFA Analysis

The quantified internal standard, normal nonadecanoic acid (19:0), was employed to dissolve the extracted and processed fatty acid methyl esters obtained from the soil samples. Gas chromatography (Agilent 7890A, Santa Clara, CA, USA) was utilized to obtain the response values for the characteristic peaks of each fatty acid region [22]. A reference to previously compiled PLFA markers [23] was used to select 19 specific types (Table 2) to characterize the structure and biomass of the soil microbial community. Additionally, the total microbial biomass (T-PLFA) for the six microbial groups was calculated, and ratios including the ratio of Gram-positive bacteria to Gram-negative bacteria (G^+^/G^−^), the environmental pressure (cy17:0c/16:1ω7c), and the ratio of fungi to bacteria (F/B) were determined.

#### 2.2.3. High-Throughput Sequencing Methods for AMF

Five grams each of soil and root samples were used for DNA extraction. A total of 1000 μL of cetyltrimethylammonium bromide (CTAB) lysate, 20 μL of lysozyme, and 1 μL of RNase A were used to extract the DNA of AMF from roots and soil according to the CTAB method [24]. There were three biological replicates for each sample. The quality of DNA extraction was analyzed using 1.2% agarose gel electrophoresis [25,26]. DNA was amplified using polymerase chain reaction (PCR) with a target fragment length of 280 bp. The AMF-specific primers AMV4.5NF (5′-AAG CTC GTA GTT GAA TTT CG-3′) and AMDGR (5′-CCC AAC TAT CCC TAT TAA TCA T-3′) were utilized to accomplish this process [27,28,29]. Gel electrophoresis was performed on the amplification products to obtain valid samples for the amplification of specific fragments. The assay conditions were a 2% gel concentration, 80 V, and 40 min of electrophoresis time.

Paired-end sequencing was performed on the samples using a sequencer (Illumina NovaSeq 6000, San Diego, CA, USA). Due to the possibility of sequencing misalignment in the raw data, splicing and filtering were performed to obtain valid data. DADA2 was employed for denoising, and sequences with an abundance of less than five were filtered out. Amplicon sequence variant (ASV) feature sequences with nearly 100% similarity were obtained through clustering [30]. Each ASV feature sequence was aligned with the Nt database to eliminate non-Glomeromycota sequences. This was followed by a BLAST comparison of the remaining representative sequences on MaarjAM (https://maarjam.botany.ut.ee/, accessed on 10 March 2023) to attribute the sequences to the AMF virtual taxa and determine the genus information [31,32]. This classification was a virtual classification based on small subunit rRNA genes in the MaarjAM database [33]. A virtual taxon was the classification unit, representing a well-supported monophyletic clade with sequence similarity within the clade exceeding a threshold of 97% [34]. Analyses including the ASV abundance, Venn diagrams, alpha diversity, and beta diversity were performed to obtain information on the absolute and relative abundances, shared and unique ASVs, and diversity indices, including the species richness (Chao1), Shannon diversity (Shannon), Simpson’s diversity (Simpson), and evenness (Pielou_e) indices [35].

### 2.3. Data Analysis

Zone mapping was performed using ArcGIS 10.8 (Environmental Systems Research Institute, Redlands, CA, USA). An independent samples *t*-test was utilized to analyze the variability of the soil microbial biomass and soil microbial community structure ratio. One-way analysis of variance (ANOVA) and Duncan’s multiple comparisons were used to complete the analysis of the AMF community data within the roots and soil of *G. biloba*, including the soil chemical, absolute, and relative abundances, shared and unique ASVs, and diversity indices such as Chao1, Shannon, Simpson, and Pielou_e. Differences were considered significant at *p* < 0.05 and highly significant at *p* < 0.01. Pearson’s coefficients were used to analyze the soil microbial biomass and correlation factors representing habitat conditions, as well as between AMF species and correlation factors representing habitat conditions. All statistical analyses were performed using SPSS version 22 (IBMSPSS Statistics, Armonk, NY, USA). These statistics were used to complete a drawing in Origin Pro 2022 (OriginLab, Hampden, MA, USA). Principal coordinate analysis (PCoA) was performed using the online platform Novogene Cloud (https://magic.novogene.com/ accessed on 27 July 2024) [36].

## 3. Results

### 3.1. Soil Environmental Traits

As displayed in Table 3, the results of soil chemical analyses indicated that there were significant differences between GYXT and XYXT. Except for the TN and OM, all other indices exhibited significant differences (*p* < 0.05). GYXT had significantly higher TN and TK contents than XYXT (*p* < 0.05), where the difference in TK was highly significant (*p* < 0.01). However, the TP, OM, pH, and electrical conductivity (EC) were lower in GYXT than in XYXT, with significant differences in the TP and EC (*p* < 0.05) and highly significant differences in pH (*p* < 0.01).

### 3.2. Microbial Biomass of the Rhizosphere Soil of G. biloba

**Figure 2:**
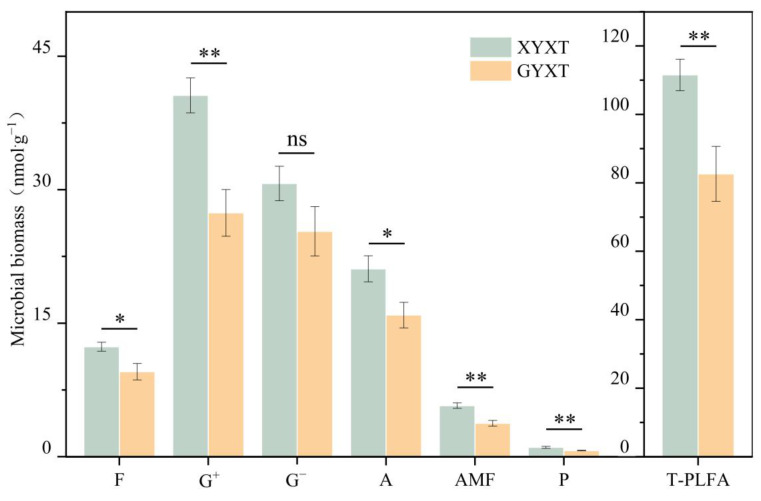
Microbial biomass in the rhizosphere soil of *Ginkgo biloba*. ns indicates *p* > 0.05, * indicates *p* < 0.05, ** indicates *p* < 0.01. The following is the same.

In total, 19 specific PLFAs were detected in the 13 soil samples examined, and the same microbial species were present in XYXT and GYXT; however, there were large differences in their contents (Figure 2). XYXT had a higher biomass of F, G^+^, G^−^, A, AMF, and P than GXYT, and all except for G^−^ were significantly different (*p* < 0.05). Among them, the biomass of G^+^, AMF, and P showed highly significant differences (*p* < 0.01). Calculating the total microbial biomass revealed that the total microbial biomass of GYXT was 82.61 nmol·g^−1^, which was highly significant (*p* < 0.01) and lower than that of XYXT. The highest content of PLFA biomarkers in XYXT and GYXT was found in G^+^, which accounted for 36.40% and 33.16% of the total PLFA biomarkers in the soils, respectively, and had the greatest predominance in the soils. The content of AMF in XYXT accounted for 5.14% of T-PLFA, which was slightly higher than that detected in GYXT (4.54%).

### 3.3. Relationship Between Soil Environmental Traits and Microbial Biomass

**Figure 3:**
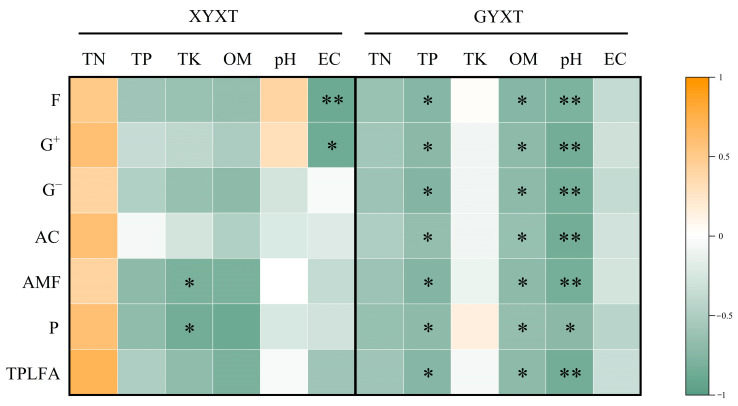
Analysis of soil environmental traits and microbial biomass. * indicates *p* < 0.05, ** indicates *p* < 0.01.

Pearson’s correlation analysis showed a significant correlation (*p* < 0.05) between the soil environmental traits and the microbial community structure. However, large differences in the soil environmental factors had significant effects on the microbial community structure in both ancient and adult *G. biloba* soils (Figure 3). In XYXT, the TK was significantly negatively correlated with AMF and P (*p* < 0.05), whereas the EC was significantly negatively correlated with F (*p* < 0.05) and significantly negatively correlated with G^+^ (*p* < 0.05). In GYXT, the TP and OM were significantly negatively correlated with F, G^+^, G^−^, AC, AMF, P, and the T-PLFA (*p* < 0.05). pH was significantly negatively correlated with P (*p* < 0.05) and highly negatively correlated with F, G^+^, G^−^, AC, AMF, and the T-PLFA (*p* < 0.01).

Fungi and bacteria are the most dominant microbial groups in soil, and the F/B and G^+^/G^−^ ratios can indirectly respond to the survival environment of soil microorganisms and the diversity characteristics of bacterial communities. As shown in Table 4, the F/B ratio of GXYT was 0.009 higher than that of XYXT, and the G^+^/G^−^ ratio was 0.246 lower than that of XYXT. The environmental pressure of GXYT was 0.360, which was significantly lower than that of XYXT (*p* < 0.05).

### 3.4. Analysis of the AMF Community in the Roots and Rhizosphere Soil of G. biloba

#### 3.4.1. Composition of the AMF Community

Abundant AMF resources were found in the roots and rhizosphere soil of *G. biloba* (Table 5). In total, 224 AMF virtual taxa were obtained from all samples using high-throughput sequencing. These taxa belonged to four orders, ten families, and nineteen genera. Among them, the genus with the most identified species was *Scutellospora*, with five species, followed by *Rhizophagus* with four species, *Paraglomus* and *Ambispora* with three species, and *Glomus*, *Septoglomus*, *Sclerocystis*, *Claroideoglomus*, and *Acaulospora* with two species. Furthermore, *Funneliformis*, *Dominikia*, *Kamienskia*, *Geosiphon*, *Diversispora*, *Redeckera*, *Gigaspora*, *Dentiscutata*, and *Pacispora* all had only one species.

The most numerous virtual taxon was *Glomus* with 137 species, accounting for 72.49% of the total virtual taxa. There were 14 species of *Paraglomus*, 12 species of *Claroideoglomus*, 10 species of *Archaeospora*, 7 species of *Acaulospora* and *Diversispora*, and 1 each of *Scutellospora*, *Ambispora*, and *Pacispora*.

#### 3.4.2. Abundance Analysis of AMF Community

**Figure 4:**
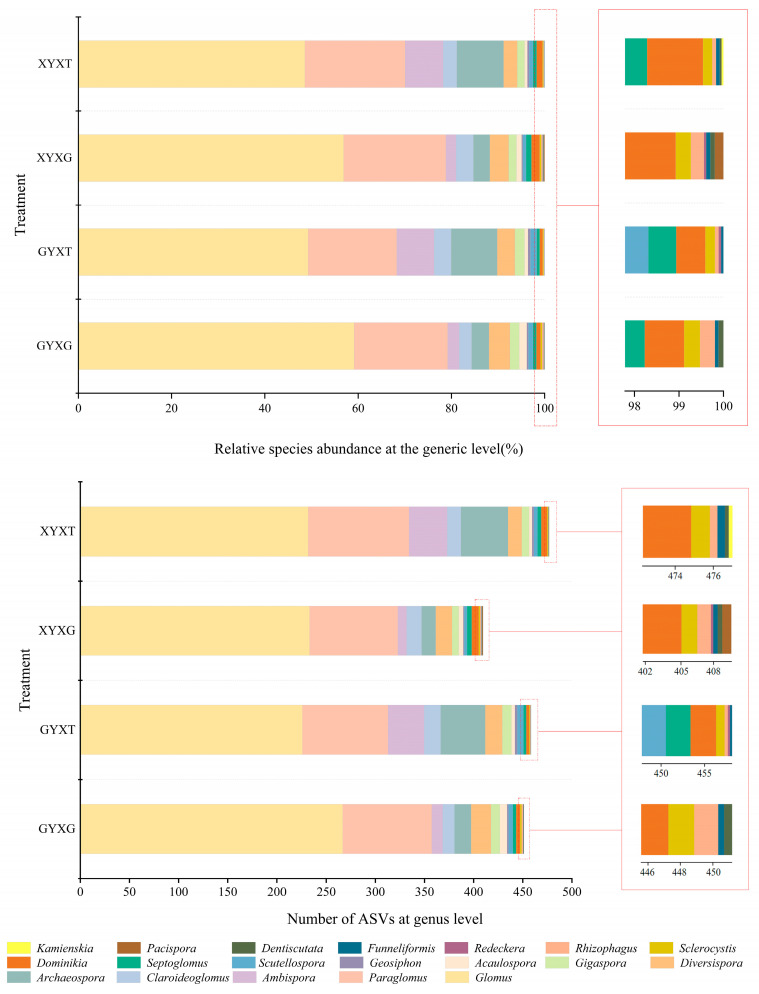
Arbuscular mycorrhizal fungi (AMF) community composition of the root and rhizosphere soil of *Ginkgo biloba* at the genus level. XYXG represents the roots of adult *Ginkgo biloba*, XYXT represents the rhizosphere soil of adult *Ginkgo biloba*, GYXG represents the roots of ancient *Ginkgo biloba*, and GYXT represents the rhizosphere soil of ancient *Ginkgo biloba*.

The genus-level composition of the AMF community in the root and rhizosphere soils of *G. biloba* is shown in Figure 4. *Glomus* was the most dominant genus in all treatments, with the number of ASVs ranging from 225 to 267 across treatments and the relative species abundance ranging from 48.55% to 59.11%. The highest ASV numbers and relative species abundances were found in GYXG, and the ASV numbers and relative species abundances of *Glomus* in the roots were higher than those in the rhizosphere soil. The second most dominant genus in all treatments was *Paraglomus*, with ASV numbers ranging from 87 to 103 and a relative species abundance between 19.01% and 21.92%. In the rhizosphere soil, the third most dominant genus was *Archaeospora*, while in the roots, the third most dominant genus was *Diversispora*. *Ambispora* was the fourth most dominant genus in GYXT and XYXT, while the fourth most dominant genera in GYXG and XYXG were *Archaeospora* and *Claroideoglomus*, respectively. Additionally, *Gigaspora*, *Acaulospora*, *Geosiphon*, *Scutellospora*, *Septoglomus*, *Dominikia*, *Sclerocystis*, *Rhizophagus*, *Funneliformis*, *Dentiscutata*, *Redeckera*, *Pacispora*, and *Kamienskia* were present in small amounts in all treatments.

#### 3.4.3. Venn Diagram Analysis of AMF Community

**Figure 5:**
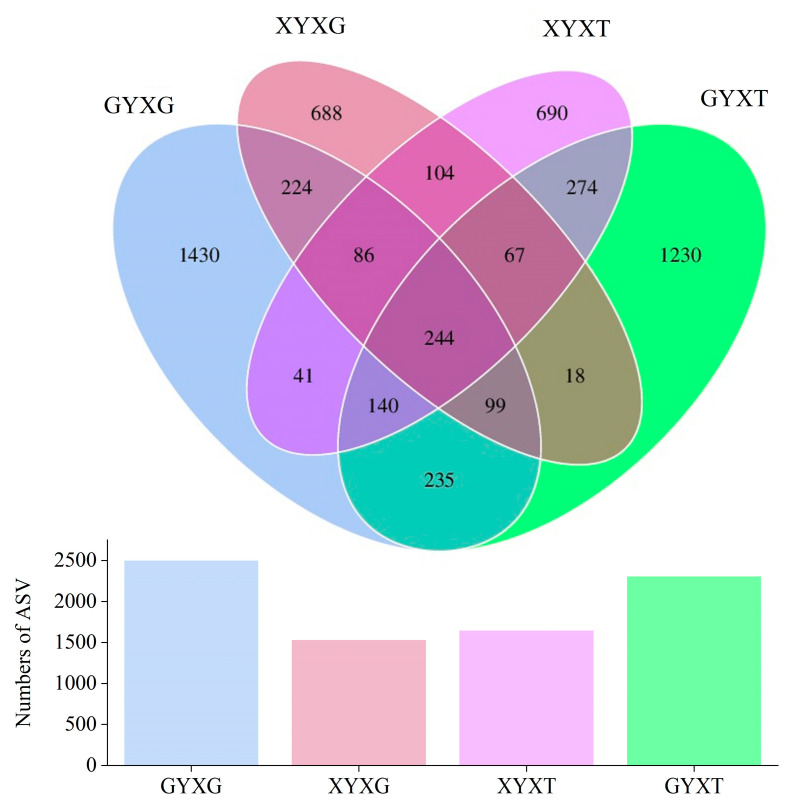
Venn diagram of arbuscular mycorrhizal fungi (AMF) community in the root and rhizosphere soil of *Ginkgo biloba*.

As shown in the Venn diagram, the numbers of total and unique ASVs in ancient *G. biloba* were greater than the corresponding ASV numbers detected in adult *G. biloba* (Figure 5). The highest numbers of total and unique ASVs in all treatments were found in GYXG, followed by GYXT and XYXT, and the lowest were found in XYXG. In addition, the number of shared ASVs in all treatments was low at only 244, accounting for 4.38% of the total number of ASVs. The highest number of shared ASVs between GYXT and XYXT was 725, and the lowest number of shared ASVs between XYXT and GYXG was 501.

#### 3.4.4. Diversity of AMF Community

**Figure 6:**
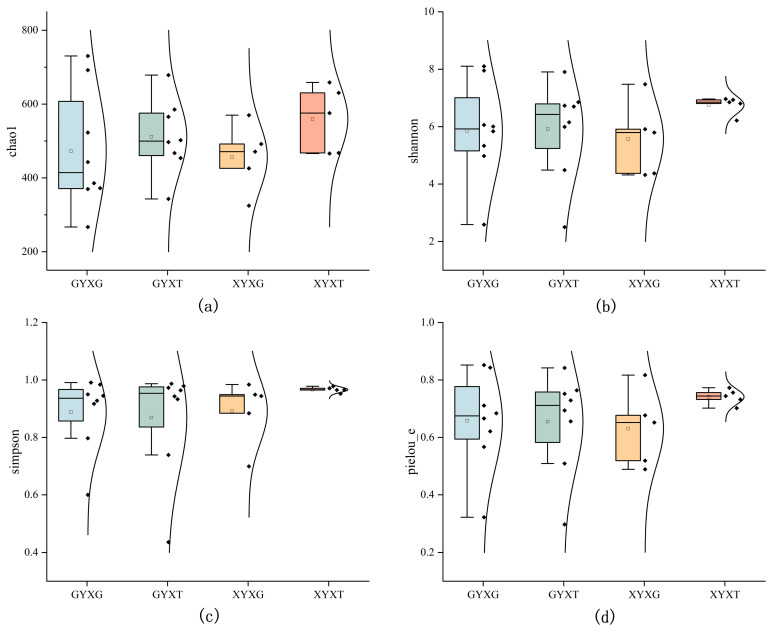
Diversity analysis of the arbuscular mycorrhizal fungi (AMF) community in the root and rhizosphere soil of *Ginkgo biloba*. (**a**) the chart of Chao1 data analysis; (**b**) the chart of Shannon analysis; (**c**) the chart of Simpson analysis; (**d**) the chart of Pielou’s evenness analysis. Each point in the graph represents the data for each sample.

The diversity of the AMF community can be assessed using diversity indices such as Shannon, Simpson, Chao1, and Pielou_e (Figure 6). As shown in Figure 6, the median Chao1 index and median Shannon index of soil samples were higher than those of the root samples. The median Simpson index of adult *G. biloba* was higher than that of ancient *G. biloba*. The Pielou’s evenness index median was highest in XYXT (0.741) and lowest in XYXG (0.631), with a slight difference of 0.003 between the ancient *G. biloba* roots and the rhizosphere soil. However, there were no significant differences in the four diversity indices among treatments. Additionally, looking at the overall picture of the four indices, the dispersion of the indices in ancient *G. biloba* was greater than that in adult *G. biloba*. The skewness was stronger, and most were negatively skewed.

#### 3.4.5. PCoA of the AMF Community

**Figure 7:**
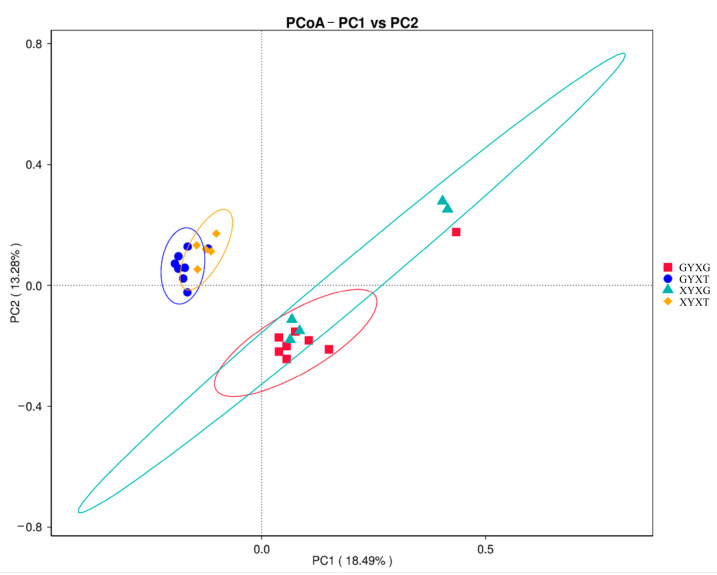
Principal coordinate analysis (PCoA) of arbuscular mycorrhizal fungi (AMF) community diversity in the root and rhizosphere soil of *Ginkgo biloba*.

PCoA using the unweighted UniFrac distance matrix algorithm revealed that the AMF community structures in the root and rhizosphere soils of *G. biloba* were significantly different (Figure 7). The AMF community structures of GYXG and XYXG were more similar, and those of GYXT and XYXT were more similar. The PCoA results indicated that a small number of widely differing biological replicates appeared in GYXG and XYXG, but all were clustered in the first and second quadrants, were positively correlated with PC1, and were basically negatively correlated with PC2. The biological replicates of GYXT and XYXT were all well clustered together, clustered in the third and fourth quadrants, were negatively correlated with PC1, and were basically positively correlated with PC2.

#### 3.4.6. Cluster Analysis of AMF Community

**Figure 8:**
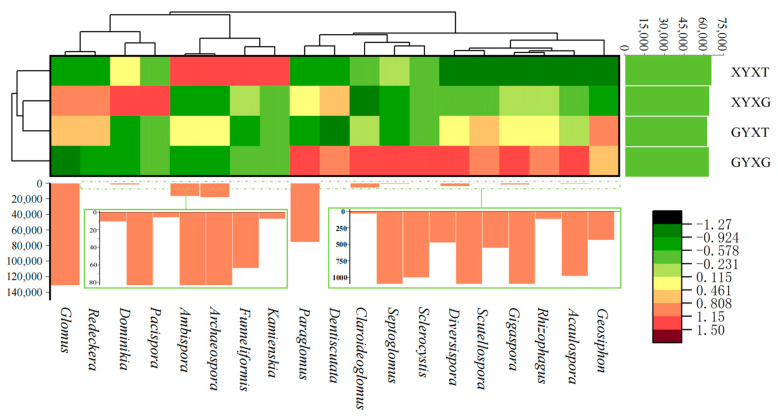
Species clustering heat map of the arbuscular mycorrhizal fungi (AMF) community in the root and rhizosphere soil of *Ginkgo biloba*.

As shown in the cluster analysis heat map, the genera with relatively high abundances varied among all treatments (Figure 8). In the root treatments, seven genera had relatively high abundances in GYXG, namely *Paraglomus*, *Claroideoglomus*, *Septoglomus*, *Sclerocystis*, *Diversispora*, *Gigaspora*, and *Acaulospora*, whereas relatively high abundances of *Dominikia* and *Pacispora* were observed in XYXG. In the soil treatments, the relatively high abundance of *Geosiphon* in GYXT differed from that in XYXT, which had relatively high abundances of *Ambispora*, *Archaeospora*, *Funneliformis*, and *Kamienskia*.

#### 3.4.7. Relationship between Soil Environmental Traits and the AMF Community

**Figure 9:**
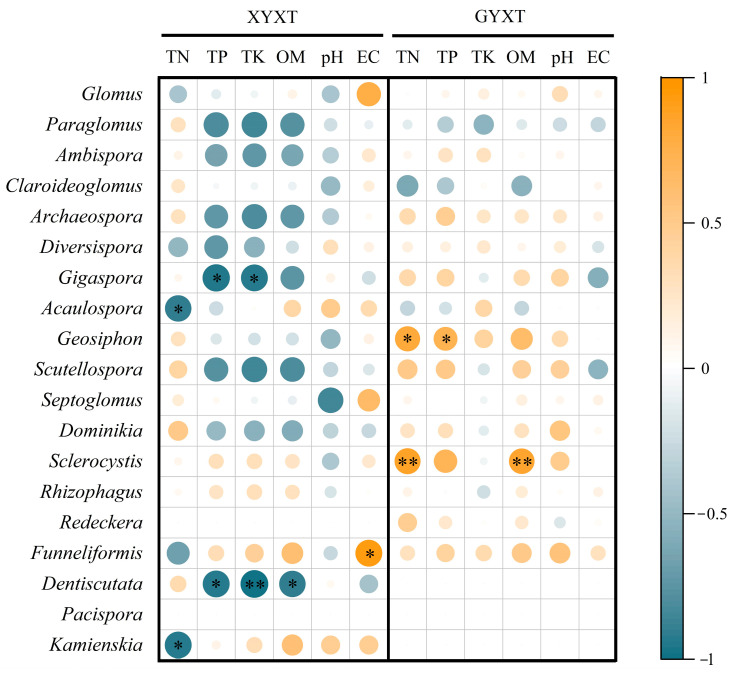
Analysis of soil environmental traits and the arbuscular mycorrhizal fungi (AMF) community. * indicates *p* < 0.05, ** indicates *p* < 0.01.

Soil environmental factors were significantly correlated (*p* < 0.05) with AMF community structure (Figure 9). The soil environmental factors that significantly affected the AMF species composition in the soils of ancient and adult *G. biloba* were quite different. In XYXT, *Gigaspora* was significantly negatively correlated with the TP and TK (*p* < 0.05), and *Dentiscutata* was significantly negatively correlated with the TN and TP (*p* < 0.05). In GYXT, *Geosiphon* was significantly positively correlated with the TN and TP (*p* < 0.05), while *Sclerocystis* was significantly positively correlated with the TN and OM (*p* < 0.01).

## 4. Discussion

Ruan et al. [7] reported a complex relationship between *G. biloba* and soil microbial communities. In the present study, similar to most tree species, bacteria contributed the greatest proportion of the microbial biomass of *G. biloba* rhizosphere soil, followed by actinomycetes and fungi, and the lowest proportion was that of protozoa. The T-PLFA in the rhizosphere soil is an indicator of microbial community richness. A high total amount represents a rich microbial community structure with a large number of microbial species and populations, which is favorable for nutrient uptake by plants [37]. In the present study, the 19 PLFAs chosen to characterize the soil microbial community structure and biomass were present in the rhizosphere soils of ancient and adult *G. biloba*, which was inconsistent with hypothesis (1). Moreover, contrary to hypothesis (1), the microbial biomass in the rhizosphere soil of ancient *G. biloba* was significantly lower than that of adult *G. biloba*. Previous studies have demonstrated that soil microbial biomass and diversity tend to decrease with increasing tree age, especially for the bacteria and actinomycetes involved in cycling soil substances. In contrast, fungi, which have lower metabolic capabilities, may increase in quantity or in terms of the proportion of the total microbial biomass [38]. The F/B ratio of ancient *G. biloba* in the present study was slightly higher than that of adult *G. biloba*, confirming this hypothesis. There is a possibility that the rhizosphere soil of ancient *G. biloba* may change from a “bacterial” to a “fungal” pattern with the increase of planting time. The F/B ratio reflects the stability of the microbial community; the larger the F/B ratio, the greater the stability [39]. This indicated that the rhizosphere soil microbial community of ancient *G. biloba* was more stable than that of adult *G. biloba*. The G^+^/G^−^ ratio reflects the soil nutrient status, where the higher the value, the higher the nutrient stress [40], suggesting that adult *G. biloba* trees experience stronger nutrient stress.

Diax et al. [41] concluded that the distribution of the soil microbial population was affected by the combination of local climatic conditions, hydrothermal conditions, soil nutrient status, soil texture, and vegetation composition. According to the correlation analysis between the soil microbial biomass and environmental factors in the present study, soil microorganisms are extremely sensitive to environmental changes, and TP, OM, and pH may be the key factors influencing the soil microbial community in the rhizosphere zone of ancient *G. biloba*. Tan et al. [42] and Lauber et al. [43] found that changes in the TP content of the soil affected the diversity of the soil bacterial and fungal communities. Accordingly, the significantly lower TP content in ancient *G. biloba* than in adult *G. biloba* may be the main reason for the lower microbial biomass in ancient *G. biloba*. In addition, the soil microbial biomass is related to the soil fertility. The rhizosphere soil of adult *G. biloba* in this study had a higher organic matter content, and the density of soil bacteria and actinomycetes was also high, which was consistent with the findings of Liu et al. [44]. Although *G. biloba* has less stringent requirements for soil pH, the metabolic processes of microorganisms have optimal pH ranges. The results of this study suggest that microorganisms in the *G. biloba* rhizosphere prefer alkaline soils. Therefore, improving the TP, OM, and pH of the soil can increase the microbial biomass in the rhizosphere soil of ancient *G. biloba*, enhance the microbial community diversity, and promote the absorption of nutrients by ancient trees.

Tong et al. [17] found that inoculation with mycorrhizal fungi could promote the growth of ancient trees and increase soil fertility in practical applications. Therefore, this study examined AMF, which contributes the largest proportion of fungal biomass, in greater depth. Cai et al. [45] also demonstrated that inoculation with AMF treatments also increased the growth and reproduction of phosphate-dissolving bacteria, fungi, and actinomycetes in the soil, but there were significant differences in the increase of the soil microbial population of different AMF. Owing to its ability to generate large amounts of amplicon data, high-throughput sequencing technology is considered one of the most effective molecular techniques for studying community structures and identifying AMF species. It is worth mentioning that this experiment is the first study to employ high-throughput sequencing technology to investigate the diversity of AMF communities in adult *G. biloba*. This is different from most applications of high-throughput sequencing technology in relation to *G. biloba* transcriptome, whole genome, and molecular genetic marker discovery [46]. The results indicated the presence of a rich diversity of AMF in *G. biloba* roots and rhizosphere soil (four orders, ten families, and nineteen genera), which were assigned to 224 virtual taxa. This provides a more comprehensive evaluation than the morphological identification results of nine species from four genera of AMF in *G. biloba* rhizosphere in Zhejiang Province. Additionally, the present study detected a higher number of ASVs in ancient *G. biloba* than in adult *G. biloba*, suggesting closer mutualistic symbiosis between ancient *G. biloba* and AMF. This aligned with the findings of Pei et al. [47], who reported an increasing trend in the number of AMF operational taxonomic units in the rhizosphere soil of *Panax notoginseng* with increasing planting years. Interestingly, this contradicted the results of the PLFA analysis, which indicated a significantly lower soil microbial biomass in the rhizosphere of ancient *G. biloba* than in the rhizosphere soil of adult *G. biloba*. This suggests that although AMF resources are abundant in the rhizosphere soil of ancient *G. biloba*, there may be an accumulation of dead but not fully decomposed AMF over long-term growth, leading to a lower biomass of live AMF.

In contrast, in terms of the AMF species composition and community structure, the situation was broadly similar in the roots and rhizosphere soil of ancient and adult *G. biloba*. The first and second most dominant genera were *Glomus* and *Paraglomus*, respectively, in the roots and rhizosphere soils of *G. biloba*, which was consistent with results reported in different ecosystems by other researchers. *Glomus*, in particular, is ecologically adaptable, extremely widespread, and a dominant genus in most ecosystems [48,49,50]. Some studies have reported that *Paraglomus* is the second most dominant genus after *Glomus* in some studies [51,52]. In contrast, *Archaeospora* and *Ambispora* were dominant in the rhizosphere, whereas *Diversispora* and *Claroideoglomus* were dominant in the roots. Furthermore, cluster analysis indicated that seven genera had a closer relationship with ancient *G. biloba* roots, namely *Paraglomus*, *Claroideoglomus*, *Septoglomus*, *Sclerocystis*, *Diversispora*, *Gigaspora*, and *Acaulospora*, whereas *Dominikia* and *Pacispora* had a closer relationship with younger adult *G. biloba* roots. These findings imply that the application of *Diversispora* and *Claroideoglomus* as mycorrhizal fungi in the rhizosphere of ancient *G. biloba* may improve the mycorrhizal infestation rate.

As evidenced by the aforementioned results, hypothesis (2) is valid. Next, the soil environments of the sampling sites were explored. Differences were found in the effects of different soil factors on AMF. In the rhizosphere soil of adult *G. biloba*, the results suggest that TN may be the main influencing factor for *Acaulospora* and *Kamienskia*, EC may be the main influencing factor for *Funneliformis*, and TP and TK may be the main influencing factors for *Gigaspora* and *Dentiscutata*. *Dentiscutata* may also be influenced by OM. *Geosiphons* may be affected by TN and TP in the rhizosphere soil of ancient *G. biloba*, *Geosiphon* may be affected by TN and TP, and *Sclerocystis* may be influenced by TN and OM. Rhizosphere soil factors are important ecological factors that have complex effects on AMF species, diversity, and abundance. However, none of the six soil factors tested had a significant effect on either *Claroideoglomus* or *Diversispora*, which can infest the roots of *G. biloba*. In future research, additional soil factors should be investigated to explore the driving factors and provide a more suitable soil environment.

Furthermore, it will be necessary to isolate and identify the dominant strains in the rhizosphere soil of ancient *G. biloba* through wet screening, cultivation, and propagation. In addition, the rhizosphere soil should be inoculated with suitable AMF agents to increase the biomass of living AMF in the rhizosphere of ancient trees and improve the mycorrhizal infestation rate and root vigor of ancient *G. biloba*. This might produce a more obvious effect than the application of exogenous fungi. It is hoped that self-produced mycorrhizal agents obtained from indigenous fungi can reduce the cost of rejuvenation and realize the great potential of using AMF as biofertilizers. This will promote the localized application of suitable AMF to ancient *G. biloba*, provide more products and choices for the protection and rejuvenation of ancient trees, and play a role in promoting the sustainable protection of ancient trees.

## Figures and Tables

**Table 1:** Overview of *Ginkgo biloba* habitat survey and soil sampling sites.

Category	Number	Tree Age/Years	Longitude and Latitude	Site	Growth Status	Growth Environment Characteristics
Ancient *G. biloba*	0214	250	31°23′3″ N, 121°14′46″ E	Liuyi Community	normal	Within the green area, there is a cluster of ancient trees, surrounded by residential areas and parking lots. There is a considerable amount of construction and household waste in the vicinity.
	0215	250			normal	
	0217	250			normal	
	0343	180			normal	
	0345	180	31°20′37″ N, 121°14′11″ E	Ziqi Donglai Park	normal	In the park, grass is regularly removed under the trees, and the surrounding area is covered with white clover vegetation.
	0346	180			normal	
	0352	180	31°21′24″ N, 121°14′6″ E	Near Shengxin Road Suspension Binhai Bridge	normal	Lakeside area with exposed ground, protected by a fence.
	0353	180			normal	
Adult *G. biloba*	YL1	50	31°9′14′′ N, 121°8′35″ E	Hua Le Road	normal	Adjacent to the main urban thoroughfare, limited growing space.
	YL2	50			normal	
	YL3	50			normal	
	YL4	50			normal	
	YL5	50			normal	

Note: The growth status of ancient *G. biloba* was judged according to the People’s Republic of China LY/T 3073-2018 “Technical regulations for the management and conservation of old and notable trees”. The growth of adult *G. biloba* was judged by the same industry standards.

**Table 2:** Characteristic microbes of phospholipid fatty acids (PLFAs).

Characterization of Microbial Taxa	PLFA Species
Fungi (F)	18:1ω9c, 18:2ω6c, and 18:3ω6c
Gram-positive bacteria (G^+^)	i14:0, i15:0, a15:0, i16:0, a17:0, and i17:0
Gram-negative bacteria (G^−^)	16:1ω7c, cy 17:0c, 18: 1ω7c, and cy 19:0c
Actinomycetes (A)	10Me16:0, 10Me17:0, and 10Me18:0
Arbuscular mycorrhizal fungi (AMF)	16:1ω5c
Protozoa (P)	20:3ω6c and 20:4ω6c

Note: i, a, cy, and Me represent isopropyl, anti-isopropyl, cyclopropyl, and methyl-branched fatty acids, respectively; ω and c represent methyl terminal and homeotropic space structures, respectively.

**Table 3:** Soil chemical analyses of the rhizosphere soil of *Ginkgo biloba*.

Soil	TN (g·kg^−1^)	TP (g·kg^−1^)	TK (g·kg^−1^)	OM (g·kg^−1^)	pH	EC (mS·cm^−1^)
XYXT	1.34 ± 0.08 a	2.02 ± 0.15 a	4.10 ± 0.32 b	30.85 ± 3.51 a	8.23 ± 0.03 a	0.17 ± 0.014 a
GYXT	2.00 ± 0.28 a	1.39 ± 0.22 b	5.78 ± 0.19 a	29.29 ± 4.32 a	7.79 ± 0.05 b	0.13 ± 0.005 b

Note: XYXT represents the rhizosphere soil of adult *Ginkgo biloba*, and GYXT represents the rhizosphere soil of ancient *Ginkgo biloba*. Different lowercase letters indicate significant differences (*p* < 0.05). The following is the same.

**Table 4:** Soil microbial community structure ratios of *Ginkgo biloba*.

Treatments	F/B	G^+^/G^−^	cy17:0c/16:1ω7c
XYXT	0.173 ± 0.004 a	1.346 ± 0.122 a	0.599 ± 0.006 a
GYXT	0.182 ± 0.004 a	1.100 ± 0.027 a	0.360 ± 0.012 b

Different lowercase letters indicate significant differences (*p* < 0.05).

**Table 5:** Virtual taxa of AMF in the root and rhizosphere soil of *Ginkgo biloba* obtained via high-throughput sequencing.

Order	Family	Genus	Virtual Taxa
Glomerales	Glomeraceae	*Glomus*	G. *microaggregatum* VTX00104, *G. hoi* VTX00199
			VTX00053, VTX00066, VTX00068, VTX00070, VTX00072–VTX00074, VTX00076, VTX00077, VTX00079, VTX00080, VTX00082–VTX00084, VTX00088, VTX00089, VTX00093, VTX00096, VTX00098, VTX00109, VTX00112, VTX00120–VTX00122, VTX00124–VTX00127, VTX00137, VTX00140, VTX00143, VTX00150, VTX00151, VTX00154, VTX00156, VTX00159, VTX00166, VTX00167, VTX00174, VTX00175, VTX00178–VTX00184, VTX00186, VTX00188, VTX00189, VTX00191, VTX00194, VTX00197, VTX00200, VTX00202, VTX00206, VTX00209, VTX00212, VTX00214, VTX00216, VTX00219, VTX00223, VTX00224, VTX00234, VTX00235, VTX00253, VTX00259, VTX00270, VTX00290–VTX00292, VTX00294, VTX00301, VTX00304, VTX00305, VTX00309, VTX00312, VTX00316, VTX00317, VTX00319, VTX00322–VTX00327, VTX00329, VTX00331, VTX00333, VTX00342–VTX00344, VTX00359, VTX00360, VTX00362, VTX00364, VTX00366, VTX00368–VTX00373, VTX00382–VTX00384, VTX00386–VTX00390, VTX00392, VTX00393, VTX00395, VTX00397–VTX00399, VTX00404, VTX00409, VTX00411–VTX00413, VTX00416, VTX00419, VTX00420, VTX00422, VTX00423, VTX00426, VTX00427, VTX00432, VTX00436, VTX00437, VTX00441–VTX00443, VTX00448, VTX00452, and VTX00453
Glomerales		*Rhizophagus*	*R. clarus* VTX00090, *R. proliferus* VTX00099, *R. intraradices* VTX00105, and *R. irregularis* VTX00114
		*Septoglomus*	*S. viscosum* VTX00063 and *S. constrictum* VTX00064
		*Sclerocystis*	*S. sinuosa* VTX00069 and *S. coremioides* VTX00268
		*Funneliformis*	*F. mosseae* VTX00067
		*Dominikia*	*D. indica* VTX00222
		*Kamienskia*	*K. perpusilla* VTX00287
	Claroideoglomeraceae	*Claroideoglomus*	*C. lamellosum* VTX00193 and *C. claroideum* VTX00225 and VTX00279
			VTX00055–VTX00057, VTX00237, VTX00276–VTX00278, VTX00297, VTX00340, VTX00357, VTX00358, and VTX00402
Paraglomerales	Paraglomeraceae	*Paraglomus*	*P. brasilianum* VTX00239, *P. laccatum* VTX00281, and *P. majewskii* VTX00335
			VTX00001, VTX00002, VTX00308, VTX00337, VTX00348–VTX00352, VTX00375, VTX00433, VTX00435, VTX00444, and VTX00446
Archaeosporales	Ambisporaceae	*Ambispora*	*A. leptoticha* VTX00242, *A. fennica* VTX00283, *A. granatensis* VTX00339
			VTX00405
Archaeosporales	Archaeosporaceae	*Archaeospora*	VTX00004–VTX00009, VTX00051, VTX00338, VTX00376, and VTX00450
	Geosiphonaceae	*Geosiphon*	*G. pyriformis* VTX00241
Diversisporales	Diversisporaceae	*Diversispora*	*D. epigaea* VTX00061
			VTX00040, VTX00306, VTX00354–VTX00356, VTX00380, and VTX00401
		*Redeckera*	*R. fulvum* VTX00262
	Acaulosporaceae	*Acaulospora*	*A. spinosa* VTX00026 and *A. longula* VTX00028
			VTX00013, VTX00015, VTX00016, VTX00227, VTX00231, VTX00272, and VTX00328
	Gigasporaceae	*Scutellospora*	*S. dipurpurescens* VTX00049, *S. nodosa* VTX00052, *S. spinosissima* VTX00254, *S. projecturata* VTX00260, and *S. nodosa* VTX00261
			VTX00318
		*Gigaspora*	*G. decipiens* VTX00039
		*Dentiscutata*	*D. heterogama* VTX00255
	Pacisporaceae	*Pacispora*	*P. scintillans* VTX00284
			VTX00011

## Data Availability

The following information was supplied regarding data availability: Data is available at NCBI BioProject accession number PR]NA1142869 (SAMN42966095, SAMN42966096, SAMN42966097, SAMN42966098, SAMN42966099, SAMN42966100, SAMN42966101, SAMN42966102, SAMN42966103, SAMN42966104, SAMN42966105, SAMN42966106, SAMN42966107, SAMN42966108, SAMN42966109, SAMN42966110, SAMN42966111, SAMN42966112, SAMN42966113, SAMN42966114, SAMN42966115, SAMN42966116, SAMN42966117, SAMN42966118, SAMN42966119, SAMN42966120.
